# “CRISPR” validation of recessive brain cancer genes *in vivo*

**DOI:** 10.18632/oncotarget.4864

**Published:** 2015-07-15

**Authors:** Marc Zuckermann, Daisuke Kawauchi, Jan Gronych

**Affiliations:** Division of Molecular Genetics, German Cancer Research Center (DKFZ), Heidelberg, Germany

**Keywords:** Chromosome Section, somatic gene transfer, CRISPR/Cas9, brain tumors, mouse models

Profound understanding of the molecular events driving oncogenic transformation is essential for the development of improved therapies against cancer. While accumulating next generation sequencing data provide a plethora of candidate genes for various cancer entities [1], validating the oncogenic potential of these genes proved to be more challenging. Autochthonous mouse models emerged as a gold standard for confirming a gene's pathogenetic relevance, since murine models feature high homology of genomic structure and broad conservation of gene function when compared with human. The generation of a genetically engineered mouse (GEM) line, however, is a time consuming process. Especially, for the analysis of multiple simultaneous gene alterations, which is an essential aspect of current cancer research, extensive breeding of multiple GEMs is often not feasible.

The genetic manipulation of somatic cells *in situ* holds great advantages in terms of speed and flexibility. While the delivery of oncogenes using e.g. viral vectors had long been established, somatic knockout of specific tumor suppressor genes has been accomplished with site-specific endonucleases including zinc-finger and transcription activator-like effector nucleases only recently. They have shown their potential to induce specific double strand breaks, possibly causing somatic mutation by error prone non-homologous end joining. Recently, the CRISPR/Cas9 system has excelled by providing the possibility to construct efficient endonucleases in a fast and convenient manner [2]. It has been shown that the somatic delivery of CRISPR nucleases via lentivirus [3] or hydrodynamic gene delivery [4] generates precise genetic alterations in specific organs and can be used to induce oncogenic transformation. Modelling of brain tumors utilizing the CRISPR technology, however, had remained to be established.

In our recent work, we aimed at extending the methodology of somatic CRISPR/Cas9-mediated gene disruption by utilizing *in utero* electroporation and *in vivo* chemical transfection [5]. These techniques are less restricted by transgene size than viral vectors, do not cause additional mutations by insertion of the transgene and can be adapted to target a variety of mouse tissues. Using these approaches, we delivered CRISPR nucleases targeting particular tumor suppressor genes in the murine brain and thereby induced specific types of brain tumors. Somatic deletion of *Ptch1* in murine cerebella induced SHH-subgroup medulloblastoma with high penetrance and short latency. These tumors resembled germline GEM models, in which *Ptch1* is genetically ablated in the cerebellum. Whole genome sequencing of these tumors facilitated an unbiased search for off targets. Utilizing this approach, we did not detect any recurrently mutated genes other than *Ptch1* in CRISPR/Cas9-induced medulloblastomas. Additionally, deletion of *Nf1*, *Pten* and *Trp53* in the murine forebrain efficiently induced glioblastoma, demonstrating that this approach is also suited to induce other brain tumor entities by targeting multiple tumor suppressor genes simultaneously. Hence, our approaches prove the feasibility of functionally validating various recessive candidate genes using the CRISPR/Cas9 system in a fast and flexible manner. Furthermore, this paves the way for more refined animal models, in which a tumor is driven by specific somatic mutations resembling what is observed in patients.

The future holds great potential in the field of CRISPR/Cas9 *in vivo* applications. First *in vivo* screens using pooled lentiviral CRISPR libraries for malignant transformation and metastasis have recently been conducted [6]. Further higher level multiplexing of these guided endonucleases is likely to be utilized leveraging somatic gene transfer approaches. With latest modifications to the CRISPR/Cas9 system, also gain-of-function mutations may be analyzed by inducing specific mutations using homology-directed repair, large genomic deletions and activation of transcription. It is conceivable that it soon will become possible to model most kinds of mutations endogenously in the mouse, allowing for highly sophisticated *in vivo* models. Together with accelerated candidate gene validation, this will greatly improve *in vivo* preclinical steps in the development of novel targeted cancer therapies.
